# Supplementary material for: Cysteine Oxidation Promotes Dimerization/Oligomerization of Circadian Protein Period 2
Source: Biomolecules. 2022 Jun 25;12(7):892. doi: 10.3390/biom12070892 (PMC9313148; doi:10.3390/biom12070892)
Supplement: Supplementary file 1 [file biomolecules-12-00892-s001.zip › biomolecules-1761824-supplementary.pdf]

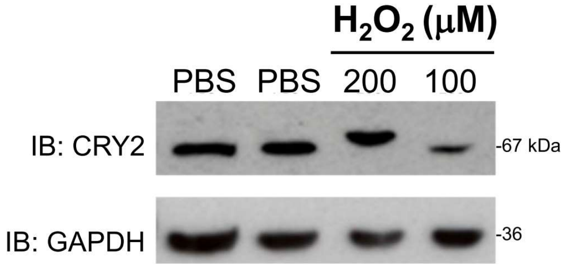

Figure S1. PEG-switch assay for CRY2. Band corresponding to CRY2 (67kDa close to 75kDa in the ladder), or GAPDH (36kDa, 37kDa in the ladder), show displacement to higher molecular weights adding 5kDa PEG, after treatment with 200 μM hydrogen peroxide.

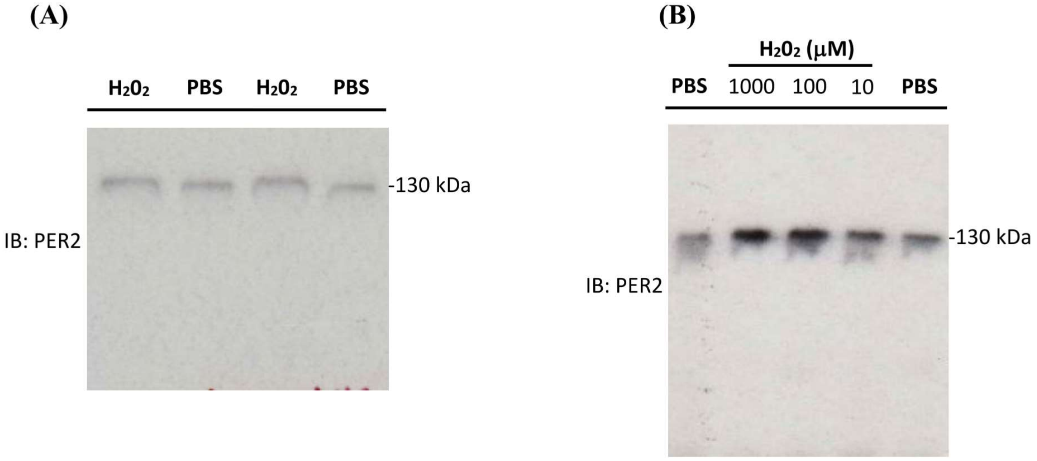

Figure S2. Western blots showing PEG Switch for PER2 assayed under different conditions. (**A**) 10kDa PEG, 8% acrylamide, 200 μM hydrogen peroxide (H202). (**B**) 20 kDa PEG, 15% acrylamide.
